# Expression of SORL1 and a novel SORL1 splice variant in normal and Alzheimers disease brain

**DOI:** 10.1186/1750-1326-4-46

**Published:** 2009-11-04

**Authors:** Karrie E Grear, I-Fang Ling, James F Simpson, Jennifer L Furman, Christopher R Simmons, Shawn L Peterson, Frederick A Schmitt, William R Markesbery, Qiang Liu, Julia E Crook, Steven G Younkin, Guojun Bu, Steven Estus

**Affiliations:** 1 Department of Physiology, Sanders-Brown Center on Aging, University of Kentucky, Lexington, KY, USA; 2 Department of Neurology, Sanders-Brown Center on Aging, University of Kentucky, Lexington, KY, USA; 3 Department of Pathology and Division of Neuropathology, Sanders-Brown Center on Aging, University of Kentucky, Lexington, KY, USA; 4 Departments of Pediatrics and Cell Biology and Physiology, Washington University School of Medicine, St Louis, MO, USA; 5 Biostatistics Unit, Mayo Clinic, Jacksonville, FL, USA; 6 Department of Neuroscience, Mayo Clinic, Jacksonville, FL, USA

## Abstract

**Background:**

Variations in sortilin-related receptor (SORL1) expression and function have been implicated in Alzheimers Disease (AD). Here, to gain insights into SORL1, we evaluated SORL1 expression and splicing as a function of AD and AD neuropathology, neural gene expression and a candidate single nucleotide polymorphism (SNP).

**Results:**

To identify SORL1 splice variants, we scanned each of the 46 internal SORL1 exons in human brain RNA samples and readily found SORL1 isoforms that lack exon 2 or exon 19. Quantification in a case-control series of the more abundant isoform lacking exon 2 (delta-2-SORL1), as well as the "full-length" SORL1 (FL-SORL1) isoform containing exon 2 showed that expression of FL-SORL1 was reduced in AD individuals. Moreover, FL-SORL1 was reduced in cognitively intact individuals with significant AD-like neuropathology. In contrast, the expression of the delta-2-SORL1 isoform was similar in AD and non-AD brains. The expression of FL-SORL1 was significantly associated with synaptophysin expression while delta-2-SORL1 was modestly enriched in white matter. Lastly, FL-SORL1 expression was associated with rs661057, a SORL1 intron one SNP that has been associated with AD risk. A linear regression analysis found that rs661057, synaptophysin expression and AD neuropathology were each associated with FL-SORL1 expression.

**Conclusion:**

These results confirm that FL-SORL1 expression declines in AD and with AD-associated neuropathology, suggest that FL-SORL1 declines in cognitively-intact individuals with AD-associated neuropathology, identify a novel SORL1 splice variant that is expressed similarly in AD and non-AD individuals, and provide evidence that an AD-associated SNP is associated with SORL1 expression. Overall, these results contribute to our understanding of SORL1 expression in the human brain.

## Background

SORL1 is a mosaic protein consisting of an amino-terminal portion resembling the vacuolar protein sorting-10 (Vps10) receptor family and a carboxyl-terminal portion having attributes of the low-density lipoprotein receptor (LDLR) family. As such, this type-1 transmembrane protein is capable of binding ligands ranging from receptor-associated protein (RAP) to apolipoprotein E (apoE) [1]. Recently, SORL1 has also been associated with AD at several levels. First, SORL1 interacts with amyloid precursor protein in the Golgi and endosomes to reduce production of the amyloid-β (Aβ) peptide, i.e., decreased SORL1 expression results in increased Aβ production *in vitro *and in a murine model *in vivo *[2-4]. Second, SORL1 expression is decreased in the neurons of sporadic AD patients [5,6], consistent with its possible role in contributing to Aβ accumulation. Furthermore, SORL1 expression is not decreased in familial AD, suggesting that diminished SORL1 expression is likely not a consequence of amyloid accumulation [7]. Third, genetic variants within SORL1 have been associated with AD in many case-control series although other studies have failed to achieve significance [8-14]. In summary, SORL1 variants that reduce SORL1 expression or function may increase AD risk by increasing Aβ production.

Alterations in RNA splicing have emerged as a major mechanism of action of functional genetic variants in diseases ranging from frontotemporal dementia to atypical cystic fibrosis to myotonic dystrophy (reviewed in [15,16]). Here, we report an analysis of SORL1 expression and splicing in AD versus non-AD brain, reporting that the expression of FL-SORL1 but not delta-2-SORL1 is associated with AD and AD neuropathology, synaptophysin expression, and an AD-associated SNP.

## Methods

### Human autopsy tissue

The University of Kentucky AD Center Neuropathology Core generously provided human brain specimens from the anterior cingulate as well as superior and middle temporal gyri. Diagnoses of AD and non-AD were performed at a consensus conference of the AD Center Neuropathology and Clinical Cores and were based upon evaluation of both cognitive status, i.e., Clinical Dementia Rating and Mini-Mental State Examination (MMSE) scores, as well as neuropathology, i.e., Braak stages which rate the extent of neurofibrillary pathology into the neocortex and NIA-Reagan Institute (NIA-RI) neuropathology classification, which includes counts of both neuritic senile plaques and neurofibrillary tangles and provides a likelihood staging of AD neuropathological diagnosis [17,18]. The age at death for individuals that were cognitively intact, i.e., non-AD, was 82 ± 9 years (mean ± SD, n = 28) while age at death for AD individuals was 82 ± 6 (n = 29). The average post-mortem interval (PMI) for non-AD individuals was 2.8 ± 0.8 hours (mean ± SD, n = 28) while the PMI for AD individuals was similar at 3.4 ± 0.6 hours (n = 29). Non-AD individuals had Mini-Mental State Examination (MMSE) scores of 28.4 ± 1.6 (n = 28). Neuropathologic evaluation by NIA-RI criteria revealed that among the 28 non-AD individuals, a total of 12 had findings consistent with no likelihood of AD (age at death of 79.8 ± 12.0 (mean ± SD)), while another 10 were categorized as having low likelihood (age at death of 84.6 ± 4.9 (n = 10), and six as moderate likelihood (age at death of 85.0 ± 3.5). The postmortem interval for individuals in each class was similar, i.e., 3.3 ± 0.7 (mean ± SD, n = 12), 2.4 ± 0.7 (n = 10), and 2.0 ± 0.4 (n = 6), respectively. Additionally, the 28 non-AD, cognitively intact individuals included 20 individuals that were Braak stages 0-II, while the remaining eight individuals were Braak stages III-V [18]. Neuropathology in AD individuals was robust, i.e., Braak stages were uniformly VI. The temporal lobe samples were prepared to compare SORL1 expression in matched white and gray matter samples. For this study, white and gray matter were carefully dissected from the temporal lobe; the quality of the separation was confirmed subsequently by a comparison of the ratio of mRNAs associated with neurons, astrocytes and oligodendrocytes, i.e., neurofilament-H, GFAP, and myelin basic protein, respectively.

### PCR amplification

Total RNA was extracted and converted to cDNA in one microgram aliquots with random hexamers and reverse transcriptase (Invitrogen, SuperScriptII) as we described previously [19-21]. Primers that spanned SORL1 cDNA were designed such that the splicing efficiency of each internal exon was evaluated (Table 1). In initial screening, pools of six AD and six non-AD cDNA samples were subjected to PCR-amplification (Platinum Taq, Invitrogen) by using each primer pair and a PCR profile consisting of pre-incubation for 2 minutes at 94°C, followed by 30 cycles of 94°C for 30 sec, 60°C for 30 sec and 72°C for 1 min (Perkin Elmer 9600). PCR products were separated by polyacrylamide gel electrophoresis, stained with SYBR-gold and visualized by using a fluorescence imager (Fuji FLA-2000). PCR products of interest were identified by direct sequencing (Davis Sequencing).

**Table 1:** SORL1 PCR primers.

**Exons Amplified**	**Primer**	**Primer Sequence**
1-5	Exon 1 Forward	GAGCTCTCTGCGAAGTCTGG
	
	Exon 5 Reverse	AAAAGGACTTGACATGTTCCTGA

4-8	Exon 4 Forward	GCCCAGTACCTCTGGATCAC
	
	Exon 8 Reverse	CTCACCAAGGTGTCACTGC

7-11	Exon 7 Forward	CATCTCTTGGGCAGTGAACA
	
	Exon 11 Reverse	CCACCTGGCTCCAGCACT

10-15	Exon 10 Forward	CCCATCCTGTCCAAGGAGT
	
	Exon 15 Reverse	AGTAGAACCCACAGGGCAAG

14-18	Exon 14 Forward	GAGTGTTTGCTGGGACACAA
	
	Exon 18 Reverse	TTTGAAGCCTGCATCTACCC

17-21	Exon 17 Forward	GTGGCCCTGGACTTTGACTA
	
	Exon 21 Reverse	CAGTGTTCTTCCCCTTGTAGAAA

20-24	Exon 20 Forward	TTACTGGACGGATGCCTACC
	
	Exon 24 Reverse	TTTCATCACTGTTGTCTCCACA

23-29	Exon 23 Forward	TGAGCGATGAGAGAAACTGC
	
	Exon 29 Reverse	TGGAAACCGAACTCATCACA

28-32	Exon 28 Forward	CGCACTTCATGGACTTTGTG
	
	Exon 32 Reverse	CCATCCTGGCAATCTTGGT

31-35	Exon 31 Forward	TGTCTGCCCAATTACTACCG
	
	Exon 35 Reverse	GTGCCTTGCTGAGACACTGA0

34-40	Exon 34 Forward	ATGCCCTCTGCTTCTTGTGT
	
	Exon 40 Reverse	ATGCTTCCTCGGAAGTTCAA

39-43	Exon 39 Forward	ATCCCACCACCAGATATCCA
	
	Exon 43 Reverse	CCTGGTCAGGAGAGTCATACG

42-46	Exon 42 Forward	ACTCCACAACAAGACGGTCA
	
	Exon 46 Reverse	CAGCTCATCGTACAGCAGGA

45-48	Exon 45 Forward	CAGCACCTGATGCCTTAAAAA
	
	Exon 48 Reverse	CATATTGCAACTCAAAGTGCATC

FL-SORL1	Exon 2 Forward	GAGGCCCAAGAGCAGTGAT

**RT-PCR Primers**		

Delta-2-SORL1	Exon 1-3 Junction Forward	CAAGGTGTACGGACAGGTGTA

Reverse Primer	Exon 4 Reverse	GCAGAAGTCAAACGTGATCC

### Real-time PCR

Primers were designed to amplify SORL1 that retained or lacked exon 2 (Table 1). Briefly, PCR product corresponding to FL-SORL1, i.e., the isoforms containing exon 2, was amplified by using a sense forward primer corresponding to sequence within exon 2 and an antisense reverse primer corresponding to sequence within exon 4. To quantify delta-2-SORL1, i.e., the isoform lacking exon 2, a sense primer corresponding to the exon 1-3 junction was used in combination with the exon 4 antisense primer (Table 1). Amplification reactions were 1 μM with respect to each primer, 1× SYBR-green Master Mix (Applied Biosystems) and the equivalent of 20 ng of template cDNA. PCR profiles consisted of a 10 minute pre-incubation period at 95°C followed by 40 cycles of 94°C for 30 s, 60°C for 30 s and 72°C for 30 sec (BioRad Chromo4). Primer specificity was ensured by separating the PCR products via polyacrylamide gel electrophoresis and SYBR-gold staining as well as melting curves at the completion of real-time PCR. Each cDNA sample was quantified in two separate reactions relative to standard curves generated with purified and quantified PCR product standards. The copy numbers of the SORL1 isoforms were normalized relative to the geometric mean of the copy numbers of hypoxanthine-guanine phosphoribosyltransferase and ribosomal protein L13A [22]. DNA samples were genotyped for rs661057 by using unlabeled PCR primers and TaqMan FAM and VIC dye-labeled MGB probes (Applied Biosystems).

### Statistical Analysis

The percentage of the delta-2-SORL1 isoform relative to total SORL1 expression was calculated as the normalized copy number of delta-2-SORL1 divided by the normalized total SORL1 copy number. Differences in gene expression as a function of AD or gray matter versus white matter were evaluated by the two sample *t*-test; differences in expression as a function of Braak stage, NIA-RI likelihood for the neuropathological diagnosis of AD, or rs661057 were analyzed by one-way analysis of variance. The association of SORL1 expression with Braak stage, synaptophysin and/or rs661057 was evaluated by using linear regression analyses (SPSS v. 17).

## Results

**Figure 1:**
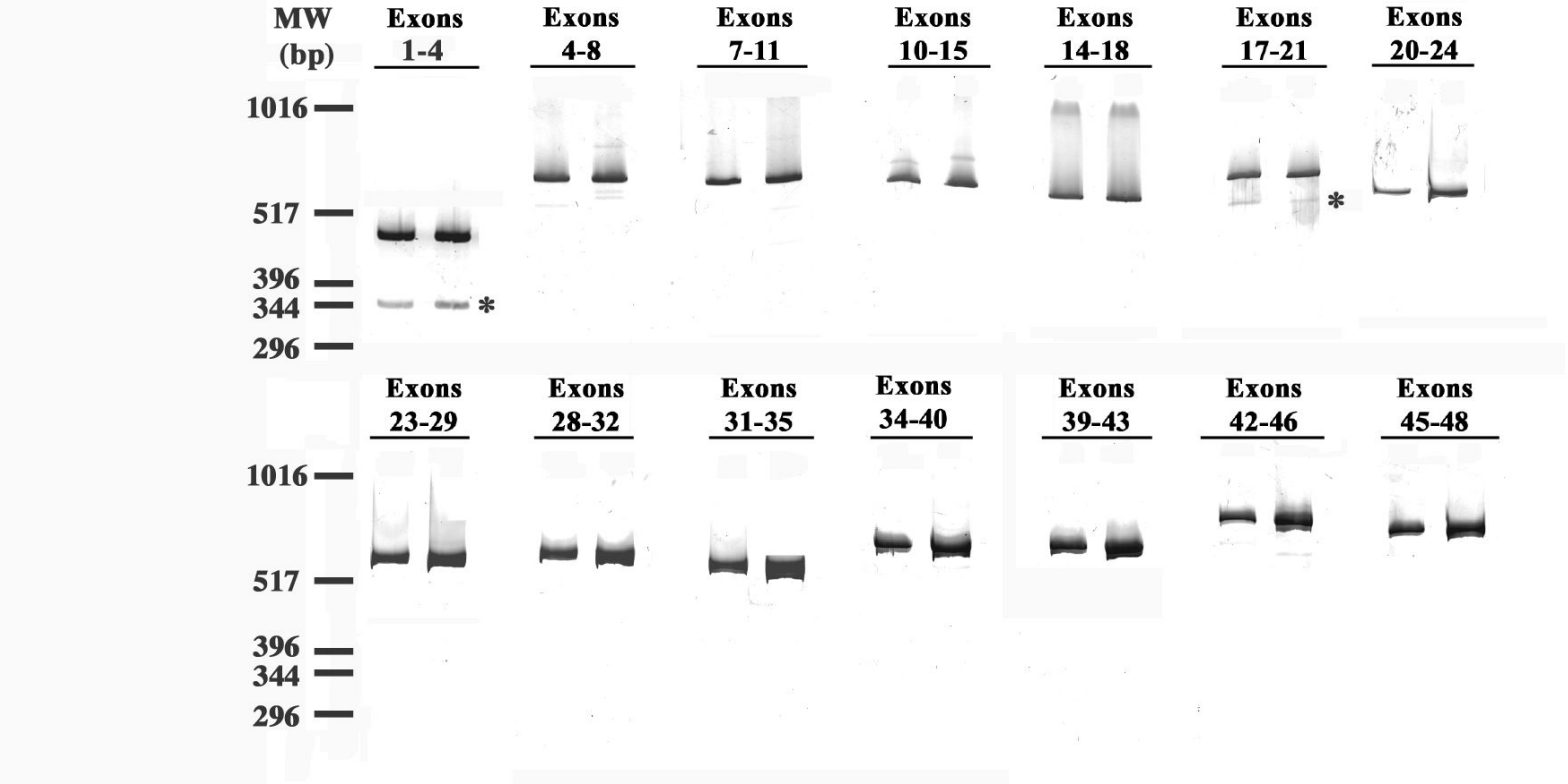
**Evaluation of SORL1 Exonic Splicing**. Inefficiently spliced exons were detected by amplifying between the indicated exons. The novel splice variants that were identified and confirmed by sequencing are marked with asterisks. Since these representative results are a montage of multiple experiments, the molecular weight markers are approximate.

**Figure 2:**
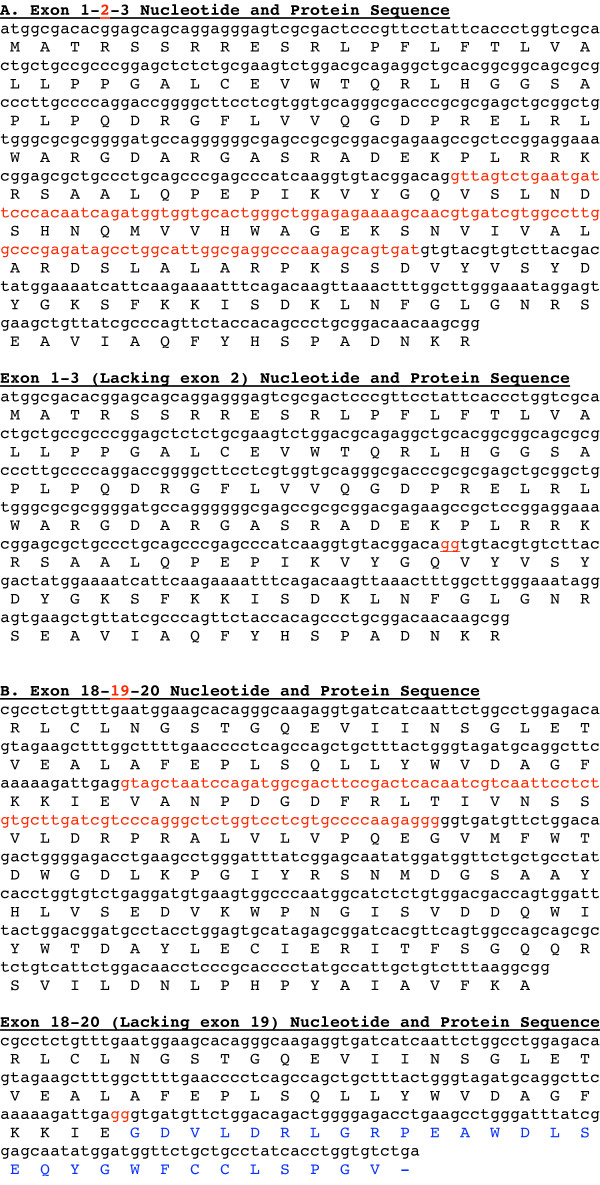
**Sequence of SORL1 Isoforms**. The nucleotide and protein sequence of FL-SORL1 as well as sequences corresponding to each of the novel isoforms is shown. Figure 2A (upper panel) depicts FL-SORL1 nucleotide and protein sequence beginning with the ATG translation start site in exon 1 through exon 3. Exon 2 is marked in red font. The nucleotide and protein sequence corresponding to delta-2-SORL1, i.e., loss of amino acids V96-D134, is shown in the lower panel, with the exon 1-3 junction indicated by red font. Figure 2B shows FL-SORL1 nucleotide and protein sequence from exon 18-20 (upper panel) with exon 19 marked in red font. The lower panel shows the nucleotide and protein sequence corresponding to delta-19-SORL1, i.e., a novel peptide sequence encoded by an out-of-frame exon 20, followed by a premature stop codon. The exon 18-20 junction is indicated by red font and the novel peptide sequence is shown in blue font.

To discern SORL1 exons that are skipped with detectable frequency, we used PCR and appropriate primer pairs to evaluate whether each of the 46 internal SORL1 exons were variably spliced in human brain cDNA. PCR products were separated by polyacrylamide gel electrophoresis and detected by SYBR-gold staining (Figure 1). SORL1 exons were generally included within the mature mRNA with high efficiency as most of the PCR reactions yielded a single product of the expected size (Figure 1). However, PCR amplification from exon 1 to exon 4 generated a 344 bp PCR product in addition to the expected 461 bp product. Similarly, the exon 17 to 21 PCR products included an unexpected 587 bp product in addition to the predicted 679 bp product. Direct sequencing of these unexpected PCR products demonstrated that the former novel product corresponded to a SORL1 isoform lacking exon 2 while the latter corresponded to a SORL1 isoform lacking exon 19. Since exon 2 does not alter the SORL1 reading frame, the amino acid sequence encoded by delta-2-SORL1 is identical to that of FL-SORL1 except for the deletion of amino acids V96 through D134 (Figure 2A). In contrast, loss of the 92 bp exon 19 leads to a shift in the codon reading frame and a premature stop codon (Figure 2B).

**Figure 3:**
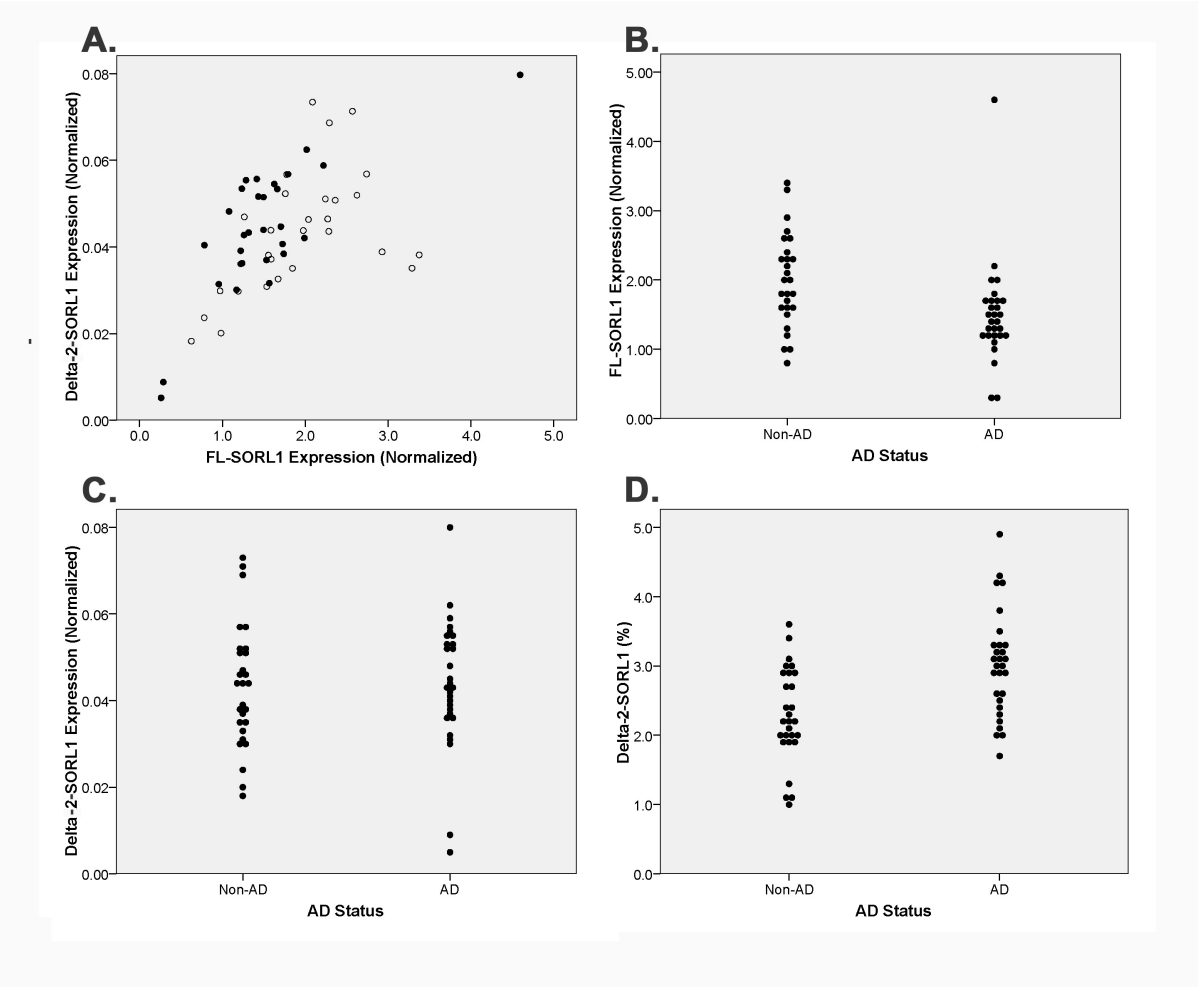
**Quantitation of FL-SORL1 and Delta-2-SORL1 in AD and non-AD Brain**. Real time PCR quantification of each isoform shows that FL-SORL1 and delta-2-SORL1 vary in parallel (A) (r^2 ^= 0.371, p < 0.001, AD individuals represented as filled circles, non-AD as open circles). FL-SORL1 was decreased in AD relative to non-AD individuals (B) while delta-2-SORL1 was similar in AD and non-AD individuals (C). Delta-2-SORL1 as a percentage of total SORL1 expression was increased in AD individuals (D).

Initial quantification of these novel SORL1 isoforms relative to the FL- SORL1 isoform suggested that delta-2-SORL1 expression represented 1-5% of total SORL1 expression while delta-19-SORL1 expression was generally less than 1% of total SORL1 expression (Figure 3, Table 2, data not shown). Hence, we focused further analyses upon the more abundant delta-2-SORL1 isoform as well as FL-SORL1. Expression of delta-2-SORL1 tended to parallel that of FL-SORL1 (Figure 3A). Evaluation of expression as a function of AD found that FL-SORL1 but not delta-2-SORL1 was decreased in AD brain samples (Figure 3B, C, Table 2). Total SORL1 expression was also significantly decreased in AD brain samples, confirming prior results [5,7], and reflecting that most of the SORL1 mRNA is FL-SORL1 (Table 2). Since the abundance of delta-2-SORL1 was relatively unchanged between AD and non-AD while FL-SORL1 was decreased, the percentage of delta-2-SORL1 was significantly increased in the AD samples (Table 2).

**Table 2:** Expression of SORL1 isoforms in AD versus non-AD brains.

**SORL1 Isoform**	**AD Diagnosis**	**N**	**Mean ± SE**	**P Value**
Total SORL1	Non-AD	28	1.978 ± 0.136	0.028
	
	AD	29	1.537 ± 0.140	

FL-SORL1	Non-AD	28	1.935 ± 0.134	0.025
	
	AD	29	1.493 ± 0.138	

Delta-2-SORL1	Non-AD	28	0.043 ± 0.003	0.868
	
	AD	29	0.044 ± 0.003	

Delta-2-SORL1 (%)	Non-AD	28	2.29 ± 0.13	<0.001
	
	AD	29	3.02 ± 0.14	

**Figure 4:**
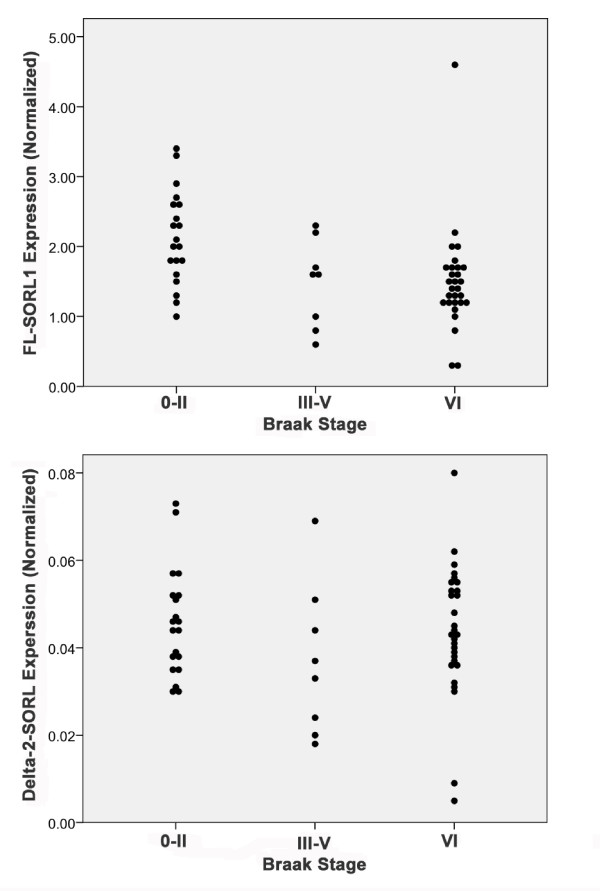
**Quantitation of FL-SORL1 and Delta-2-SORL1 as a Function of Braak Stage**. The expression of FL-SORL1 was reduced in cognitively intact individuals with a Braak stage indicative of some AD neuropathology relative to cognitively intact individuals with little neuropathology (A). In contrast, the expression of delta-2-SORL1 was similar among the groups (B).

In considering these results further, we recognized that while the non-AD individuals were all cognitively intact within a year of their death, e.g., their MMSE scores were 28.4 ± 1.6 (n = 28, range 24-30), a subset of these individuals had significant AD-associated neuropathology at autopsy. Hence, we explored whether the AD-associated decline in SORL1 expression correlated with neuropathology. First, we considered SORL1 expression relative to Braak staging, which reflects the severity of neurofibrillary pathology in the medial temporal lobe structure and extension into the cortex. Eight of the 28 non-AD individuals had significant AD-associated neuropathology, i.e., Braak stages of III, IV or V. When SORL1 expression was evaluated as a function of Braak stage (Figure 4, Table 3), individuals who were cognitively intact but had moderate AD-like pathology (Braak stages of III-V) were found to have SORL1 expression levels more similar to the AD individuals (Braak VI) than the cognitively intact individuals with little AD-like pathology (Braak stages 0-II). Hence, even among cognitively intact individuals, the expression of FL-SORL1 but not delta-2-SORL1 was reduced as the severity of AD-like neuropathology increased. We additionally evaluated SORL1 expression as a function of NIA-RI criteria, which includes both CERAD neuritic plaque scores and Braak staging. With these criteria, 22 of the non-demented individuals had neuropathology consistent with no or low likelihood of AD, while six had sufficient pathology to rank as intermediate to high AD probability, based solely on neuropathology. The expression of FL-SORL1 but not delta-2-SORL1 was again significantly decreased in the intermediate-high AD likelihood individuals, relative to the individuals with no-low likelihood of AD (Table 4). Hence, the expression of FL-SORL1 but not delta-2-SORL1 is decreased as a function of AD neuropathology.

**Table 3:** Expression of SORL1 isoforms relative to Braak stage.

**SORL1 ISOFORM**	**Braak**	**N**	**Mean ± SE**	P Value*
Total SORL1	0-II	20	2.167 ± 0.150	0.009
	
	III-V	8	1.507 ± 0.228	0.031*
	
	VI	29	1.537 ± 0.140	0.004*

FL-SORL1	0-II	20	2.121 ± 0.150	0.008
	
	III-V	8	1.470 ± 0.222	0.031*
	
	VI	29	1.493 ± 0.138	0.003*

Delta-2-SORL1	0-II	20	0.046 ± 0.003	0.339
	
	III-V	8	0.037 ± 0.006	
	
	VI	29	0.044 ± 0.003	

Delta-2-SORL1 (%)	0-II	20	2.26 ± 0.16	0.002
	
	III-V	8	2.48 ± 0.15	0.454*
	
	VI	29	3.02 ± 0.14	0.001*

For each isoform, the initial p value is the overall ANOVA p value, while the p values marked with asterisks refer to LSD post-hoc comparisons with the Braak 0-II group.

**Table 4:** Expression of SORL1 isoforms in human brain as a function of NIA-RI neuropathology classification.

**SORL1 Isoform**	**NIA-RI**	**N**	**Mean ± SE**	**P Value**
Total SORL1	Zero-Low	22	2.023 ± 0.151	0.033
	
	Mod-High	35	1.584 ± 0.128	

FL-SORL1	Zero-Low	22	1.980 ± 0.150	0.031
	
	Mod-High	35	1.540 ± 0.126	

Delta-2-SORL1	Zero-Low	22	0.043 ± 0.003	0.826
	
	Mod-High	35	0.044 ± 0.003	

Delta-2-SORL1 (%)	Zero-Low	22	2.25 ± 0.15	0.001
	
	Mod-High	29	2.93 ± 0.13	

**Figure 5:**
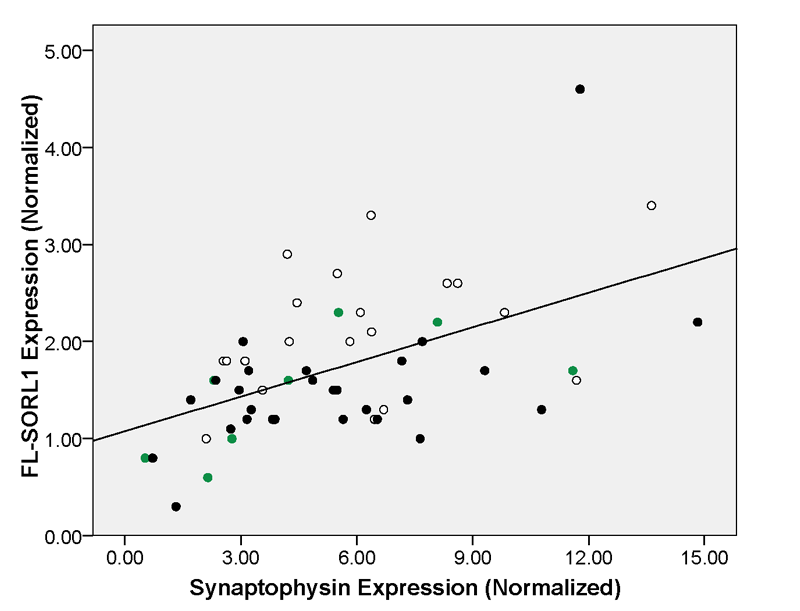
**Quantitation of FL-SORL1 as a function of synaptophysin expression and Braak stage**. Individuals that have Braak stages that are low (0-II, open circles), moderate (Braak III-V, green circles) or high (VI, black-filled circles) are indicated. Linear regression analyses found that both synaptophysin and Braak stage (two degrees of freedom) were significantly associated with SORL1 expression, (p < 0.001 and p = 0.015, respectively). Synaptophysin expression showed only a slight trend towards decreased expression with Braak stage, which did not achieve significance (p = 0.55, ANOVA)

We hypothesized that delta-2-SORL1 expression is enriched in glia because SORL1 expression was reported to be reduced only in neurons in AD and delta-2-SORL1 was not reduced in AD [5]. To assess this possibility, we quantified FL-SORL1 and delta-2- SORL1 in matched gray and white matter samples carefully dissected from five non-AD brains. We found that the percentage of delta-2-SORL1 was significantly greater in white matter than gray matter, i.e., 5.0 ± 0.7% versus 3.8 ± 0.5%, respectively (mean ± SD, n = 5, p = 0.006), consistent with the possibility that the proportion of the delta-2-SORL1 isoform varies as a function of cell type. We also evaluated SORL1 expression by using a linear regression model that included the expression of synaptophysin, which encodes a synaptic vesicle protein, as well as AD neuropathology, as reflected by Braak stage. We found that the expression of FL-SORL1 was associated with both Braak stage and synaptophysin expression (Figure 5, p = 0.015 and <0.001, respectively, adjusted r^2 ^= 0.347) while delta-2-SORL1 was not significantly associated with either factor (adjusted r^2 ^= 0.036, data not shown). In summation, the decline in FL-SORL1 expression with Braak stage is significant even when synaptophysin expression is considered as well.

**Figure 6:**
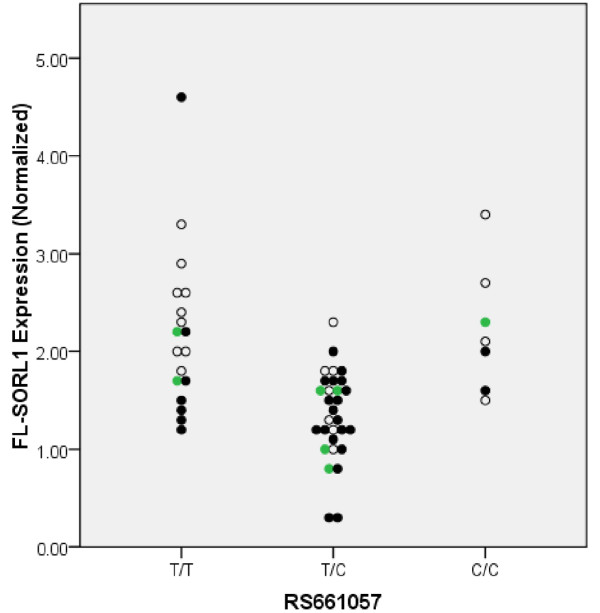
**Quantitation of FL-SORL1 as a function of rs661057 and Braak stage**. Individuals that have Braak stages that are low (0-II, open circles), moderate (Braak III-V, green circles) or high (VI, black-filled circles) are indicated.

Lastly, we evaluated whether rs661057 was associated with FL-SORL1 expression or the proportion of delta-2-SORL1; we chose this SNP because rs661057 is within intron 1 of SORL1 and has been associated with AD in some populations [8]. Rs661057 *per se *was significantly associated with the expression of both FL-SORL1 and delta-2-SORL1 (Table 5); interestingly, this association was the result of rs661057 heterozygotes having lower SORL1 expression. However, rs661057 was not associated significantly with the percentage of SORL1 that was expressed as delta-2-SORL1 and hence this SNP appeared associated with SORL1 expression *per se *in these samples. Further evaluation of the data indicated that the rs661057 heterozygotes were over-represented among the individuals with higher Braak stages (Figure 6). To discern whether the apparent rs661057 association with SORL1 expression was independent of AD neuropathology and synaptophysin expression, we analyzed FL-SORL1 expression by using a linear regression model that included rs661057 genotype, synaptophysin expression and Braak stage; since FL-SORL1 expression was similar for individuals with moderate and high Braak stages, these two categories were combined to optimize statistical power. This analysis found that the inclusion of rs661057 produced a more robust model of the data than Braak stage and synaptophysin alone, with an adjusted r^2 ^of 0.461. Braak stage, rs661057, and synaptophysin were each significantly associated with FL-SORL1 (Table 6).

**Table 5:** Expression of SORL1 isoforms in human brain as a function of rs661057 genotype.

**SORL1 ISOFORM**	**Rs661057**	**N**	**Mean ± SE**	P Value*
Total SORL1	T/T	18	2.249 ± 0.196	<0.001
	
	T/C	30	1.383 ± 0.086	<0.001*
	
	C/C	8	2.081 ± 0.295	0.552*

FL-SORL1	T/T	18	2.199 ± 0.195	<0.001
	
	T/C	30	1.344 ± 0.084	<0.001*
	
	C/C	8	2.033 ± 0.292	0.553*

Delta-2-SORL1	T/T	18	0.050 ± 0.003	0.017
	
	T/C	30	0.039 ± 0.002	0.007*
	
	C/C	8	0.048 ± 0.007	0.706*

Delta-2-SORL1 (%)	T/T	18	2.36 ± 0.20	0.072
	
	T/C	30	2.88 ± 0.14	
	
	C/C	8	2.46 ± 0.28	

For each isoform, the initial p value is the overall ANOVA p value, while the p values marked with asterisks refer to LSD post-hoc comparisons with the rs661057 T/T group.

**Table 6:** Estimated Marginal Means for FL-SORL1 Expression.

**Variable**	**Estimated Marginal** **Mean ± SE**	**P Value**
**Braak Stage**		

Low (0-II)	2.058 ± 0.127	0.046

High (III-VI)	1.706 ± 0.109	

		

**Rs661057**		

T/T	2.091 ± 0.137	0.012

T/C	1.557 ± 0.112	

C/C	1.998 ± 0.194	

These data reflect the values for the indicated parameter with synaptophysin expression normalized to 5.48. Synaptophysin expression was also significantly associated with FL-SORL1 expression (p < 0.001).

## Discussion

The primary findings of this report are four-fold. First, the expression of FL-SORL1 and total SORL1 is reduced in the AD brain, confirming prior reports [5,6]. Second, the expression of FL-SORL1 and total SORL1 is decreased in cognitively intact individuals with moderate AD neuropathology, consistent with the possibility that SORL1 declines early in the disease. Third, we report the identification of novel SORL1 isoforms lacking exons 2 or 19. Quantification of the delta-2-SORL1 isoform reveals that this isoform is expressed at similar levels in AD and non-AD individuals. Lastly, we interpret our data as suggesting a model for SORL1 expression that includes AD neuropathology, synaptophysin expression, and rs661057, an AD-associated SNP. Overall, these results provide insight into variables associated with SORL1 expression and show that exon skipping is a rare event in SORL1 mRNA.

Since the first report of reduced SORL1 expression in AD neurons in 2004 [5], a predominant theory has emerged that a reduction in functional SORL1 contributes to increased Aβ and, thereby, increased AD risk [2,6-8]. Here, we confirm prior results showing that SORL1 expression is generally reduced in AD [5,6]. Additionally, we report that SORL1 expression is reduced in individual that were cognitively intact but yet had moderate AD-like pathology, and hence could represent "preclinical" AD subjects [23,24]. This result is similar to that of Sager et al. [6] who reported that SORL1 expression in individuals with mild cognitive impairment (MCI) was quite variable such that SORL1 expression in some MCI individuals was similar to normal individuals while SORL1 expression in other MCI individuals was reduced to levels similar to those seen in AD. Hence, the decreased SORL1 expression that we observed here in cognitively intact individuals with moderate AD neuropathology is suggestive that declines in SORL1 expression presage the onset of AD dementia.

To investigate the role of SORL1 splicing in SORL1 variation, we evaluated each of the SORL1 exons to identify those that are frequently not present within mature SORL1 mRNA. This led to the identification of the delta-2-SORL1 and delta-19-SORL1 isoforms. The former was modestly enriched in white matter relative to gray matter, consistent with its lack of association with synaptophysin expression. The latter was present at levels too low for reliable quantitation in many of the samples, and hence we were unable to compare expression among groups. The function of the proteins encoded by these novel isoforms is not currently known. Delta-2-SORL1 encodes a SORL1 protein variant that lacks amino acids V96-D134. The functional consequences of loss of this portion of the Vps10p domain are not known but could impact sorting properties of the protein. The loss of exon 19 in delta-19-SORL1 introduces a codon frameshift, resulting in an isoform that encodes the normal SORL1 protein until amino acid 857, followed by a novel 29 amino acid sequence and a premature stop codon after amino acid 886 (Figure 2). Hence, this truncated receptor is predicted to contain the intact Vsp10p domain, as well as the first two LDLR class B repeats but lack several LDLR class A repeats, the fibronectin type III domain, the transmembrane domain and the cytosolic tail. Although the function of this truncated, delta-19-encoded soluble receptor is not yet clear, Jacobsen et al. evaluated a SORL1 minireceptor that consisted of the 731 amino-terminal SORL1 amino acids and found that this truncated receptor bound RAP but not apoE [1]. A similar function may be found for delta-19-SORL1. In summation, while the function of protein encoded by delta-2-SORL1 is unclear, we speculate that the protein encoded by delta-19-SORL1 may represent a dominant negative form of SORL1 that binds some SORL1 ligands, but, since it lacks the cytosolic tail, will not modulate their sorting. If either of these SORL1 variants represent loss of function for SORL1, they may contribute to increased Aβ production and, thereby, AD risk.

In evaluating factors that associate with SORL1 expression, we arrived at a model that includes (i) AD-associated neuropathology, as reflected by Braak stage, (ii) neuronal gene expression, as reflected by synaptophysin expression, and (iii) a SORL1 intron 1 SNP, rs661057, that has been associated with AD in at least some series [8]. The association between synaptophysin and SORL1 expression likely reflects neuronal expression of SORL1 [5]. The mechanisms underlying the association between SORL1 expression and Braak stage or rs661057 are unknown. Regarding rs661057, individuals that are heterozygous for this SNP had lower SORL1 expression than individuals homozygous for the major or minor allele. Hence, rs661057 would appear to show homozygote dominance, with an unclear underlying mechanism. Consideration of the association between rs661057 and AD does not clarify this situation (meta-analysis at ,[25]) because the SNP seems to show a genotype dose dependent association with AD. Evaluation of the widespread reproducibility of rs661057 association with SORL1 expression in other studies will be of interest to the field.

## Conclusion

In summary, we confirmed that SORL1 expression is reduced in the AD brain. Moreover, SORL1 expression was decreased as a function of AD-associated neuropathology prior to dementia, suggesting that the SORL1 reduction precedes conversion to AD. The exons of SORL1 were generally included within the mature message with high efficiency, with only exons 2 and 19 being skipped at detectable levels. Delta-2-SORL1 was modestly enriched in white matter and unchanged in AD. Lastly, Braak stage, synaptophysin expression, and rs661057 were all found to be associated with SORL1 expression.

## Abbreviations

(LDLR): includes low-density lipoprotein receptor; (RAP): receptor-associated protein; (apoE): apolipoprotein E; (PMI): post-mortem interval; (MMSE): mini-mental state exam; (NIA): National Institute on Aging; (RI): Reagan Institute; (MCI): mild cognitive impairment.

## Competing interests

The authors declare that they have no competing interests.

## Authors' contributions

KG and JF initiated the project by screening for SORL1 splice variants, I-FL quantified delta-2-SORL1 and FL-SORL1, JFS quantified HPRT and RPL housekeeping genes, SP worked to quantify delta-19-SORL1, CRS performed the white/gray matter analysis, WRM performed the neuropathology dissections and interpreted neuropathology, FAS provided insights into cognition associations and data analyses, QL and GB quantified synaptophysin expression, JEC and SGY provided statistical insights, and SE supervised the overall project. All authors have read and approved the final manuscript.

## References

[B1] Jacobsen L, Madsen P, Jacobsen C, Nielsen MS, Gliemann J, Petersen CM (2001). Activation and functional characterization of the mosaic receptor SorLA/LR11. J Biol Chem.

[B2] Andersen OM, Reiche J, Schmidt V, Gotthardt M, Spoelgen R, Behlke J, von Arnim CA, Breiderhoff T, Jansen P, Wu X, Bales KR, Cappai R, Masters CL, Gliemann J, Mufson EJ, Hyman BT, Paul SM, Nykjaer A, Willnow TE (2005). Neuronal sorting protein-related receptor sorLA/LR11 regulates processing of the amyloid precursor protein. Proc Natl Acad Sci USA.

[B3] Offe K, Dodson SE, Shoemaker JT, Fritz JJ, Gearing M, Levey AI, Lah JJ (2006). The lipoprotein receptor LR11 regulates amyloid beta production and amyloid precursor protein traffic in endosomal compartments. J Neurosci.

[B4] Dodson SE, Andersen OM, Karmali V, Fritz JJ, Cheng D, Peng J, Levey AI, Willnow TE, Lah JJ (2008). Loss of LR11/sorLA enhances early pathology in a mouse model of amyloidosis: Evidence for a proximal role in Alzheimer's disease. J Neurosci.

[B5] Scherzer CR, Offe K, Gearing M, Rees HD, Fang G, Heilman CJ, Schaller C, Bujo H, Levey AI, Lah JJ (2004). Loss of apolipoprotein E receptor LR11 in Alzheimer disease. Arch Neurol.

[B6] Sager KL, Wuu J, Leurgans SE, Rees HD, Gearing M, Mufson EJ, Levey AI, Lah JJ (2007). Neuronal LR11/sorLA expression is reduced in mild cognitive impairment. Ann Neurol.

[B7] Dodson SE, Gearing M, Lippa CF, Montine TJ, Levey AI, Lah JJ (2006). Lr11/sorLA expression is reduced in sporadic Alzheimer disease but not in familial Alzheimer disease. J Neuropathol Exp Neurol.

[B8] Rogaeva E, Meng Y, Lee JH, Gu Y, Kawarai T, Zou F, Katayama T, Baldwin CT, Cheng R, Hasegawa H, Chen F, Shibata N, Lunetta KL, Pardossi-Piquard R, Bohm C, Wakutani Y, Cupples LA, Cuenco KT, Green RC, Pinessi L, Rainero I, Sorbi S, Bruni A, Duara R, Friedland RP, Inzelberg R, Hampe W, Bujo H, Song YQ, Andersen OM, Willnow TE, Graff-Radford N, Petersen RC, Dickson D, Der SD, Fraser PE, Schmitt-Ulms G, Younkin S, Mayeux R, Farrer LA, St George-Hyslop P (2007). The neuronal sortilin-related receptor Sorl1 is genetically associated with Alzheimer disease. Nat Genet.

[B9] Lee JH, Chulikavit M, Pang D, Zigman WB, Silverman W, Schupf N (2007). Association between genetic variants in sortilin-related receptor 1 (Sorl1) and Alzheimer's disease in adults with Down syndrome. Neurosci Lett.

[B10] Li Y, Rowland C, Catanese J, Morris J, Lovestone S, O'Donovan MC, Goate A, Owen M, Williams J, Grupe A (2008). Sorl1 variants and risk of late-onset Alzheimer's disease. Neurobiol Dis.

[B11] Minster RL, DeKosky ST, Kamboh MI (2008). No association of Sorl1 SNPs with Alzheimer's disease. Neurosci Lett.

[B12] Lee JH, Cheng R, Honig LS, Vonsattel JP, Clark L, Mayeux R (2008). Association between genetic variants in Sorl1 and autopsy-confirmed Alzheimer disease. Neurology.

[B13] Meng Y, Lee JH, Cheng R, St George-Hyslop P, Mayeux R, Farrer LA (2007). Association between Sorl1 and Alzheimer's disease in a genome-wide study. Neuroreport.

[B14] Tan EK, Lee J, Chen CP, Teo YY, Zhao Y, Lee WL (2009). Sorl1 haplotypes modulate risk of Alzheimer's disease in Chinese. Neurobiol Aging.

[B15] Wang GS, Cooper TA (2007). Splicing in disease: Disruption of the splicing code and the decoding machinery. Nat Rev Genet.

[B16] Orengo JP, Cooper TA (2007). Alternative splicing in disease. Adv Exp Med Biol.

[B17] (1997). Consensus recommendations for the postmortem diagnosis of Alzheimer's disease. The National Institute on Aging, and Reagan Institute working group on diagnostic criteria for the neuropathological assessment of Alzheimer's disease. Neurobiol Aging.

[B18] Nelson PT, Braak H, Markesbery WR (2009). Neuropathology and cognitive impairment in Alzheimer disease: A complex but coherent relationship. J Neuropathol Exp Neurol.

[B19] Chomczynski P, Sacchi N (1987). Single-step method of RNA isolation by acid guanidinium thiocynate-phenol-chloroform extraction. Anal Biochem.

[B20] Aksenov MY, Tucker HM, Nair P, Aksenova MV, Butterfield DA, Estus S, Markesbery WR (1999). The expression of several mitochondrial and nuclear genes encoding the subunits of electron transport chain enzyme complexes, cytochrome c oxidase, and NADH dehydrogenase, in different brain regions in Alzheimer's disease. Neurochem Res.

[B21] Zhu H, Tucker HM, Grear KE, Simpson JF, Manning AK, Cupples LA, Estus S (2007). A common polymorphism decreases low-density lipoprotein receptor exon 12 splicing efficiency and associates with increased cholesterol. Hum Mol Genet.

[B22] Vandesompele J, De Preter K, Pattyn F, Poppe B, Van Roy N, De Paepe A, Speleman F (2002). Accurate normalization of real-time quantitative RT-PCR data by geometric averaging of multiple internal control genes. Genome Biol.

[B23] Schmitt FA, Davis DG, Wekstein DR, Smith CD, Ashford JW, Markesbery WR (2000). "Preclinical" AD revisited: Neuropathology of cognitively normal older adults. Neurology.

[B24] Price JL, McKeel DW, Buckles VD, Roe CM, Xiong C, Grundman M, Hansen LA, Petersen RC, Parisi JE, Dickson DW, Smith CD, Davis DG, Schmitt FA, Markesbery WR, Kaye J, Kurlan R, Hulette C, Kurland BF, Higdon R, Kukull W, Morris JC (2009). Neuropathology of nondemented aging: Presumptive evidence for preclinical Alzheimer disease. Neurobiol Aging.

[B25] Bertram L, McQueen MB, Mullin K, Blacker D, Tanzi RE (2007). Systematic meta-analyses of Alzheimer disease genetic association studies: The Alzgene database. Nat Genet.

